# Identification of potentially deleterious mutations in gastric cancer using patient-derived xenograft models

**DOI:** 10.3389/fgene.2025.1571535

**Published:** 2026-01-29

**Authors:** Luke Kong, Jie Wang, Junqi Zheng, Xihua Yang, Ruifang Sun, Jiahui Kou, Yujie Yao, Feng Li, Fuhua Wang, Sutang Guo

**Affiliations:** 1 Basic Medical College, Shanxi Medical University, Taiyuan, Shanxi, China; 2 Department of Medical Laboratory, Jincheng People’s Hospital, Jincheng, Shanxi, China; 3 Laboratory Animal Center, Shanxi Province Cancer Hospital/Shanxi Hospital Affiliated to Cancer Hospital, Chinese Academy of Medical Sciences/Cancer Hospital Affiliated to Shanxi Medical University, Taiyuan, Shanxi, China; 4 Department of Tumor Biobank, Shanxi Province Cancer Hospital/Shanxi Hospital Affiliated to Cancer Hospital, Chinese Academy of Medical Sciences/Cancer Hospital Affiliated to Shanxi Medical University, Taiyuan, Shanxi, China; 5 Central Laboratory, Shanxi Province Cancer Hospital/Shanxi Hospital Affiliated to Cancer Hospital, Chinese Academy of Medical Sciences/Cancer Hospital Affiliated to Shanxi Medical University, Taiyuan, Shanxi, China; 6 Department of Laboratory Medicine, Shanxi Province Cancer Hospital/Shanxi Hospital Affiliated to Cancer Hospital, Chinese Academy of Medical Sciences/Cancer Hospital Affiliated to Shanxi Medical University, Taiyuan, Shanxi, China

**Keywords:** bioinformatics tools, deleterious mutations, gastric cancer, patient-derived xenografts (PDX), whole-exome sequencing (WES)

## Abstract

**Background:**

This study aimed to identify novel mutations associated with the progression of gastric cancer by establishing patient-derived xenograft (PDX) models and performing comprehensive genomic characterization of these PDX models and their corresponding primary tumors.

**Methods:**

Fresh gastric cancer tissue samples were collected from 20 patients who underwent surgical resection at Shanxi Cancer Hospital and were subsequently implanted into NOD-SCID mice to establish PDX models. Histopathological features were evaluated using hematoxylin and eosin (H&E) staining. Whole-exome sequencing (WES) was performed on both primary tumors and their corresponding F1-PDX and F3-PDX tumors, focusing on mutations within 559 cancer-related genes. Predictive tools, including SIFT, Polyphen2_HVAR, Polyphen2_HDIV, and Mutation Taster, were utilized to identify potentially deleterious mutations, while I-Mutant and MUpro were employed to assess protein stability.

**Results:**

Nine gastric cancer PDX models were successfully established, with seven models propagated to the third generation (F3-PDX), achieving an initial engraftment success rate of 45%. The latency of tumor establishment significantly decreased with each successive generation. The histological characteristics of the primary tumors were well preserved in the PDX models. WES of the three selected models revealed key mutated genes in primary tumors (F0), including *IRS2, BLM, PDE4DIP, NUMA1, MYH9, TP53, PIK3CD, ERCC5*, and *ASXL1*. A total of 28 somatic mutations were conserved across all three generations (F0, F1-PDX, and F3-PDX) in these models, representing a conservation rate of 43.75% (28/64). Among these conserved mutations, 10 were identified as potentially deleterious by multiple bioinformatics algorithms. Mutations in *PTPRK* (p.L988S), *PIK3CB* (p.F934L), *LRP1B* (p.A1912T), and *IGF2R* (p.G2052R) were predicted to significantly decrease protein stability.

**Conclusion:**

This study demonstrated that PDX models effectively preserve the biological and genetic characteristics of primary gastric tumors, underscoring their utility in studying tumor heterogeneity. The integrated analysis of longitudinal WES data from primary tumors and matched PDXs enabled the identification of a core set of conserved, potentially deleterious mutations. The four prioritized mutations (PTPRK, PIK3CB, LRP1B, and IGF2R) provide new insights into the genetic landscape of gastric cancer and represent promising candidates for the development of targeted therapeutic strategies.

## Highlights

- Successful establishment and longitudinal propagation of gastric cancer patient-derived xenograft (PDX) models in NOD-SCID mice, with histological features preserved across passages (F0 → F3).
- Integrated whole-exome sequencing (WES) analysis of matched primary tumors (F0) and PDXs (F1/F3) from three patients, revealing a core set of 28 somatic mutations conserved across all generations.
- Identification and prioritization of four novel, potentially deleterious missense mutations—*PTPRK* (p.L988S), *PIK3CB* (p.F934L), *LRP1B* (p.A1912T), and *IGF2R* (p.G2052R)—predicted to disrupt protein stability and key oncogenic pathways in gastric cancer.

## Introduction

Gastric cancer remains one of the most common malignancies worldwide, with over one million new cases diagnosed annually. Despite advances in early detection and multimodal therapy, it continues to be a leading cause of cancer-related mortality, responsible for 768,793 deaths globally in 2020 (Sung et al., 2021; Chandarana et al., 2024). The clinical management of advanced disease is particularly challenging due to pronounced inter- and intra-tumoral heterogeneity and frequent resistance to conventional chemotherapy (Cho, 2020; Marin et al., 2016). Targeted therapies represent a promising strategy to overcome these limitations by exploiting tumor-specific molecular targets. To date, however, trastuzumab—approved for first-line treatment of HER2-positive advanced gastric cancer—is the only targeted agent with established clinical utility (Zhu et al., 2021). Its efficacy is often compromised by primary or rapidly acquired resistance, and broader molecularly guided approaches have yet to translate into substantial survival gains for most patients (Wang et al., 2022; Reddavid et al., 2021). These shortcomings highlight an urgent need to deepen our understanding of gastric cancer genomics and to identify novel, actionable driver alterations.

Patient-derived xenograft (PDX) models, generated by direct engraftment of tumor tissue into immunodeficient mice, have emerged as powerful preclinical tools that faithfully recapitulate the histopathological architecture, genomic landscape, and therapeutic responses of original patient tumors across serial passages (Meehan, 2019). In gastric cancer, PDX models have been successfully employed for drug screening and molecular profiling (Peille et al., 2020; Zhu et al., 2015). Nevertheless, their potential to uncover evolutionarily conserved, potentially deleterious somatic mutations—particularly those driving clonal adaptation during engraftment and propagation—remains underexploited. Whole-exome sequencing (WES) enables comprehensive characterization of coding-region mutations, facilitating the discovery of driver genes, elucidation of mutational processes, and identification of therapeutic targets (Nemtsova et al., 2020).

Here, we established a cohort of gastric cancer PDX models and performed WES on primary tumors (F0) and their corresponding early- (F1) and late-passage (F3) PDX derivatives. Our goal was to identify a core set of somatically mutated genes conserved across passages—thereby enriched for functionally relevant alterations—and to prioritize novel, potentially deleterious variants with implications for gastric cancer pathogenesis and targeted therapy development.

## Materials and methods

### Human gastric cancer tissue specimens

Fresh gastric cancer tissue samples were collected from 20 patients who underwent surgical resection at Shanxi Cancer Hospital in 2018. All procedures were conducted in accordance with approved protocols, and written informed consent was obtained from all participants. The study protocol and tissue collection procedures were reviewed and approved by the Medical Ethics Committee of Shanxi Cancer Hospital (Approval No.202009).

### Immune-deficient mice

Female NOD-SCID (non-obese diabetic severe combined immunodeficiency) mice, aged 6–7 weeks, were purchased from Beijing Vital River Laboratory Animal Technology Co., Ltd. (Beijing, China). All animals were housed in a specific pathogen-free facility at the Laboratory Animal Center of Shanxi Cancer Hospital. All animal experiments were performed in compliance with institutional ethical guidelines and were approved by the Experimental Animal Welfare Ethics Review Committee of Shanxi Cancer Hospital (Approval No.202009).

### Establishment of gastric cancer PDX models

Fresh gastric cancer tissue specimens (designated as F0) were collected immediately after surgical resection and immersed in ice-cold DMEM. Tissues were minced into fragments of approximately 2–3 mm^3^ and surgically implanted into the subcutaneous space of the right or left axilla of recipient NOD-SCID mice. Tumor fragments from each patient were engrafted into 3–4 recipient mice, which constituted the first passage (F1) cohort. Tumor volumes were measured weekly using digital caliper and calculated according to the formula: volume = 0.5× (width^2^ × length) (Faustino-Rocha et al., 2013). Successful engraftment was defined as the formation of a measurable tumor reaching a volume of ≥1,500 mm^3^ within 180 days post-implantation.

**FIGURE 1:**
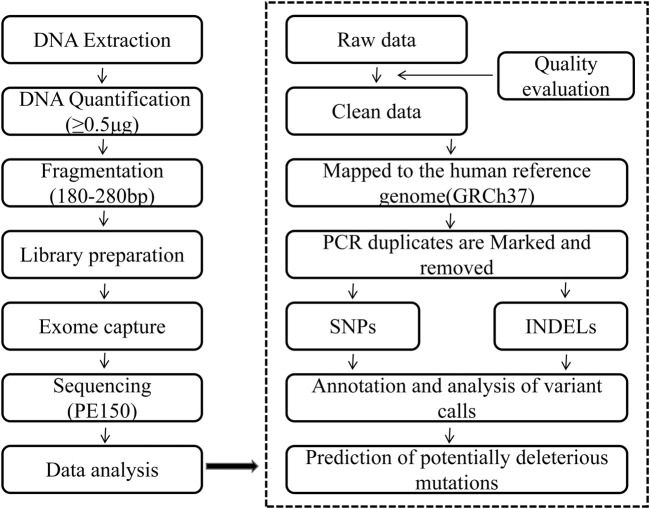
Workflow for whole-exome sequencing (WES) and bioinformatics analysis. The left panel illustrates the experimental pipeline: genomic DNA was extracted from tumor samples, quantified (≥0.5 μg), fragmented to 180–280 bp, and used for library preparation. Exome enrichment was performed using capture-based kits, followed by paired-end sequencing (PE150) on a next-generation sequencing platform. The right panel depicts the computational workflow: raw sequencing reads underwent quality control (QC) assessment and trimming to generate clean data, which were then aligned to the human reference genome (GRCh37). PCR duplicates were marked and removed, and variants—including single-nucleotide polymorphisms (SNPs) and insertions/deletions (INDELs)—were called and annotated. Functional impact of nonsynonymous variants was predicted using tools such as SIFT and PolyPhen-2, with potentially deleterious mutations identified based on combined prediction Scores.

Upon reaching predefined endpoint (tumor volume ≥1,500 mm^3^), F1 tumors were harvested and sectioned into uniform fragments (about 2–3 mm^3^). A portion of each fragment was serially passaged into new recipient mice to generate subsequent generations (F2, F3, etc.), while the remainder was cryopreserved in liquid nitrogen for downstream molecular analyses (Figure 1). Differences in tumor latency among passages were assessed using the Kruskal–Wallis test with Dunn’s *post hoc* multiple comparisons test.

### Histological analysis

Primary tumors (F0), F1-PDX, and F3-PDX tissues were fixed in 4% paraformaldehyde for 24 h. Following fixation, tissues were processed, embedded in paraffin, and sectioned at 4 μm. Sections were stained with hematoxylin and eosin (H&E) and examined under a optical microscope. Histopathological concordance between primary tumors and matched PDXs was independently assessed by two board-certified pathologists blinded to sample identity, based on three criteria: 1) tissue architecture, 2) cellular morphology, and 3) nuclear atypia. Each feature was scored on a 4-point ordinal scale: 0, no similarity; 1, mild similarity; 2, moderate similarity; 3, near-identical appearance. Initial scoring was performed independently, and any discrepancies were resolved by consensus.

### Whole-exome sequencing (WES) of 559 cancer-related genes

Among the successfully established PDX models, we selected a subset for longitudinal WES based on the following criteria: (i) robust engraftment and propagation to at least the F3 generation; (ii) high-quality DNA yield (>0.5μg, concentration ≥20 ng/μL) from both F0 and PDX tissues; and (iii) high histopathological fidelity to the original tumor.

Genomic DNA was extracted from frozen F0, F1-PDX, and F3-PDX tissues using a standard phenol-chloroform or commercial kit method, and quantified with a Qubit 2.0 fluorometer (Thermo Fisher Scientific). Samples with a DNA concentration of at least 20 ng/μL and a total yield of 0.5 μg or more were selected for library preparation. Genomic DNA was sheared to a target size of 180–280 bp using a Covaris M220 focused-ultrasonicator (Covaris, Inc.). Indexed sequencing libraries were prepared and enriched using the Agilent SureSelect XT Custom Target Enrichment Kit (Agilent Technologies) following the manufacturer’s instructions. Libraries were hybridized with biotinylated RNA baits designed to capture all exons of 559 cancer-associated genes (Supplementary Table S1). Magnetic beads coated with streptavidin were used to capture biotinylated probe–DNA hybrids. Captured libraries were PCR-amplified, and library quality was assessed using a Bioanalyzer (Agilent Technologies). Libraries were sequenced on an Illumina HiSeq PE150 platform using 150-bp paired-end reads (Illumina, Inc., San Diego, CA, USA). An average sequencing depth of ≥100× was achieved across all samples, with >99% of the target region covered at ≥20× (Figure 1).

### Sequencing data analysis

Raw sequencing reads were assessed for quality using FastQC (v0.11.9) and trimmed to remove adapters and low-quality bases using Trim Galore (v0.6.6). For PDX samples, murine stromal reads were removed using Xenome (v1.0.1) with GRCh37 (human) and GRCm38 (mouse) reference genomes. High-quality reads were aligned to the human reference genome (hg19/GRCh37) using BWA-MEM (v0.7.17) (Li and Durbin, 2009). PCR duplicates were marked and removed using Sambamba (v0.7.1) (Tarasov et al., 2015). Somatic single-nucleotide variants (SNVs) and small insertions/deletions (indels) were called using GATK Mutect2 (v4.1.20) following the GATK Best Practices workflow.

A Panel of Normals (PON) was generated from 12 in-house non-tumor gastric mucosal samples and publicly available WES data from East Asian individuals, and used in GATK Mutect2 to filter out germline variants and technical artifacts. Only variants with a read depth ≥20× and VAF ≥5% in at least one sample were retained for downstream analysis. Variant calls were annotated using ANNOVAR (2019Oct24) and converted to Mutation Annotation Format (MAF). Cross-sample comparison of mutational profiles across F0, F1-PDX, and F3-PDX was performed using the maftools R package (v2.10.0) (Mayakonda et al., 2018).

### Bioinformatics analysis

Kyoto Encyclopedia of Genes and Genomes (KEGG) pathway enrichment analysis was performed using the DAVID bioinformatics database (v6.8; https://david.ncifcrf.gov/). Enrichment significance was assessed using the hypergeometric test, and p-values were adjusted for multiple testing using the Benjamini–Hochberg procedure to control the false discovery rate (FDR). Pathways with an FDR <0.05 were considered significantly enriched.

Variant allele frequencies (VAF) across passages was visualized as a heatmap using the ggplot2 package (v3.4.0) in R (v4.3.1). For genes with multiple somatic variants, only the variant with the highest VAF in each sample was retained to represent the gene-level alteration, under the assumption that the dominant clone most likely drives functional consequences. Variants with VAF <0.1 were excluded from visualization to minimize the influence of potential sequencing artifacts. A red–white color scale was used (white: VAF = 0; dark red: VAF = 1). Samples were ordered chronologically (F0 → F1 → F3), and genes were sorted alphabetically. Plot parameters were optimized for clarity in publication.

The functional and clinical relevance of mutations was assessed by cross-referencing the dbSNP (build 155) and COSMIC (v99) databases. The functional impact of missense variants was predicted using SIFT (Kumar et al., 2009), Polyphen-2 HDIV and HVAR (Adzhubei et al., 2010), Mutation Taster (Schwarz et al., 2010), I-Mutant (Capriotti et al., 2005), and MUpro (Cheng et al., 2006).

## Results

### Establishment of gastric cancer patient-derived xenograft (PDX) models

Patient-derived xenograft (PDX) models of gastric cancer were established by subcutaneously implanting tumor fragments from 20 patients into 3–4 NOD-SCID mice per donor specimen. A total of 9 F1, 8 F2, and 7 F3 PDX lines were successfully established. Clinical characteristics of the 9 patients whose tumors yielded F1 PDX models are summarized in Supplementary Table S2.

**FIGURE 2:**
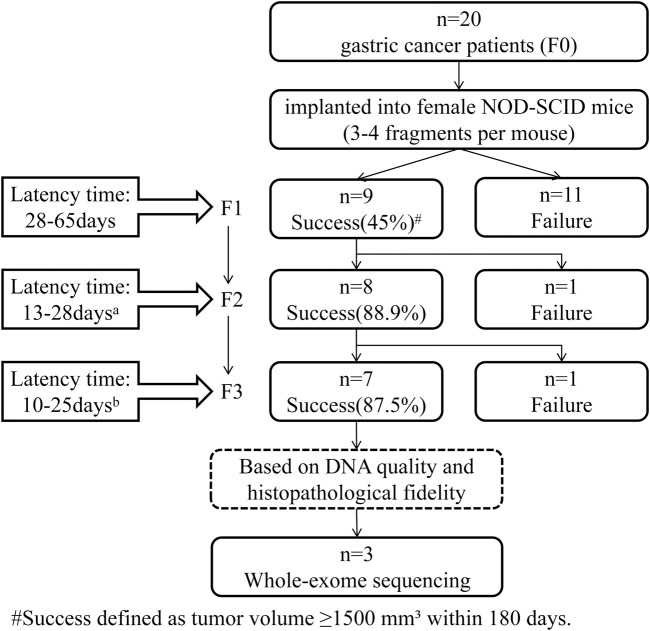
Flowchart of patient-derived xenograft (PDX) model establishment in gastric cancer. Primary tumors from 20 gastric cancer patients (F0) were implanted into female NOD-SCID mice (3–4 fragments per mouse). Engraftment success was defined as tumor volume ≥1,500 mm^3^ within 180 days. Of the initial 20 cases, 9 (45%) successfully engrafted at F1; 8 of these 9 (88.9%) propagated to F2, and 7 of 8 (87.5%) progressed to F3. The latency period—the time from implantation to tumor volume reaching ≥1,500 mm^3^—significantly decreased across passages: F1, 28–65 days; F2, 13–28 days (p = 0.003 vs. F1); F3, 10–25 days (p = 0.012 vs. F2), as determined by Dunn’s test with Benjamini–Hochberg correction for multiple comparisons. Three PDX lines were selected for whole-exome sequencing based on high DNA quality, sufficient yield, and histopathological fidelity (>80% tumor cellularity confirmed by H&E staining).

Engraftment success rates were 45% (9/20) for F0-to-F1, 88.9% (8/9) for F1-to-F2, and 87.5% (7/8) for F2-to-F3 passages. Tumor growth kinetics accelerated significantly across passages, with median time to reach 1,500 mm^3^ decreasing from 28 to 65 days in F1 to 10–25 days in F3. These intergenerational differences were statistically significant overall (Kruskal–Wallis test, p < 0.001), with pairwise comparisons revealing significant reductions in latency between F1 and F2 (p = 0.003) and between F2 and F3 (p = 0.012) (Figure 2).

**FIGURE 3:**
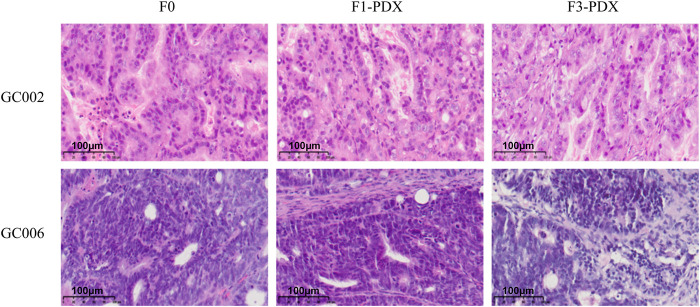
Hematoxylin and eosin (H&E) staining of primary F0 tumors and their corresponding F1-PDX and F3-PDX models from two representative cases (GC002 and GC006). Histological features—including irregular glandular architecture, branching luminal spaces (GC002), and marked nuclear atypia (enlarged, hyperchromatic nuclei with irregular contours)—are consistently preserved across generations. In GC002, the F3-PDX shows increased cellular density and reduced glandular differentiation, possibly reflecting clonal selection during serial passaging; however, nuclear morphology remains faithful to the original tumor. In GC006, tubular gland formation and nuclear pleomorphism are maintained throughout multiple passages. These findings demonstrate high morphological fidelity between primary tumors and PDX models, confirming that key histopathological characteristics are retained over successive engraftments. This preservation supports the use of these models in preclinical studies of gastric cancer biology and therapy. Scale bars: 100 μm (×20 magnification).

Histopathological assessment demonstrated high morphological concordance between primary tumors and their matched PDX models at both early (F1) and late (F3) passages. In both GC002 and GC006 PDX lines, key architectural and cytological features of the original tumors were preserved, with similarity scores consistently ranging from 2 to 3 across all three histological domains: tissue architecture, cellular morphology, and nuclear atypia (Figure 3; Supplementary Table S3). These results indicate that PDX models retain the histological features of their parental tumors across multiple passages, supporting their utility as preclinical models for translational research.

### Somatic mutations in primary gastric tumors (F0)

**FIGURE 4:**
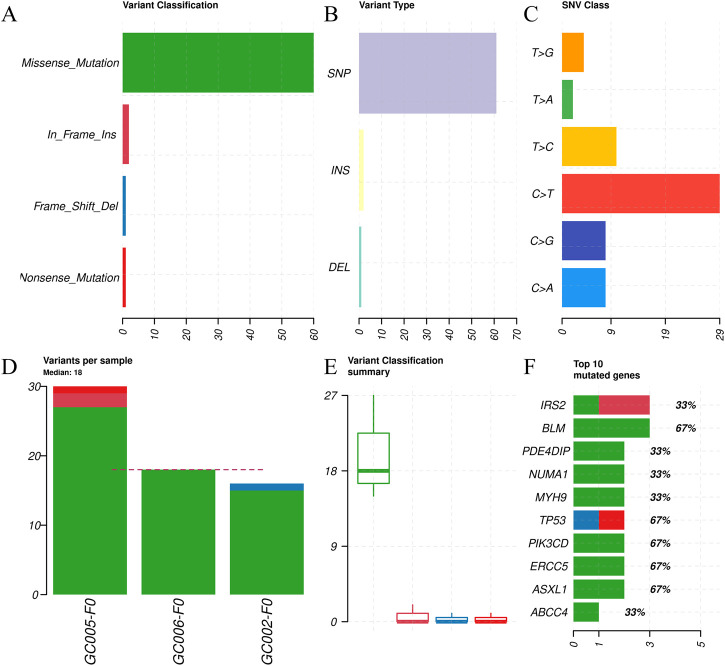
Molecular characterization of somatic mutations in primary gastric tumors based on whole-exome sequencing of 559 cancer-related genes. **(A)** Variant classification distribution: missense mutations (green) are the most frequent, followed by nonsense (light red), in-frame insertions (dark red), and frameshift deletions (blue). Missense variants account for the majority of coding alterations, reflecting their common role in functional modulation. **(B)** Distribution of variant types: single-nucleotide polymorphisms (SNPs; purple) constitute ∼60% of all variants, with insertions (INS; yellow) and deletions (DEL; cyan) occurring less frequently. **(C)** Spectrum of single-nucleotide variants (SNVs): C>T transitions (red) are the most prevalent substitution type, consistent with known mutational signatures in gastric cancer, followed by T>C (yellow), C>G (blue), and T>A (green) changes. **(D)** Number of somatic variants per tumor sample: each bar represents an individual tumor (GC002-F0, GC006-F0, GC005-F0), with the dashed line indicating the median variant burden (18 variants/sample). **(E)** Summary of variant classifications across samples: box plot showing the distribution of somatic variant counts by type. Missense mutations exhibit the highest median count, while frameshift deletions and nonsense mutations are rare. **(F)** Top 10 most frequently mutated genes in primary tumors: horizontal bar chart displaying total somatic variant counts per gene (X-axis), with color-coded mutation types (red = frameshift, green = missense). Notable recurrently altered genes include *IRS2, BLM, PDE4DP, NUMA1,* and *TP53*. In panels **(A)**, **(B)**, **(C)**, and **(F)**, the X-axis represents somatic variant counts; in panel **(D)**, the Y-axis shows variant counts per sample; in panel **(E)**, the Y-axis indicates variant counts by classification.

Whole-exome sequencing (WES) of primary tumors (F0) identified 64 somatic mutations across 53 of the 559 targeted cancer-related genes. Mutation annotation revealed that missense variants constituted the predominant mutation type (Figures 4A,E), Single-nucleotide polymorphisms (SNPs) were the most frequent class (Figure 4B), and C>T transitions represented the most common substitution (Figure 4C). Among the cohort, GC005-F0 exhibited the highest mutational burden (Figure 4D).

Nine genes harbored two or more somatic mutations across the cohort: *IRS2, BLM, PDE4DIP, NUMA1, MYH9, TP53, PIK3CD, ERCC5,* and *ASXL1*. Notably, recurrent mutations in *TP53, ERCC5, PIK3CD, BLM,* and *ASXL1* were observed in multiple tumors (Figure 4F; Supplementary Table S4).

In GC002-F0, *NUMA1* carried two missense mutations (p.R1667C and p. A794G), and *MYH9* harbored two missense variants (p.V615A and p. D7N). In GC005-F0, *IRS2* harbored three distinct in-frame indels (p.H29_N30delinsNH, p. N23_T24delinsTN) and a missense variant (p.G11V), while *PDE4DIP* carried two missense mutations (p.P30S and p. P2355H) (Supplementary Table S4).

To investigate the biological relevance of the 53 mutated genes, KEGG pathway enrichment analysis was performed (Supplementary Table S5). Of these, 40 genes were mapped to 89 KEGG pathways. Notably, 13 of these genes were annotated to the PI3K–Akt signaling pathway: *PDGFRA, CSF3R, SYK, FLT3, PIK3CD, PIK3CB, MTOR, PIK3CG, ERBB3, KIT, ERBB2, FGFR4,* and *TP53*.

### Somatic mutations in patient-derived xenografts (PDX)

**FIGURE 5:**
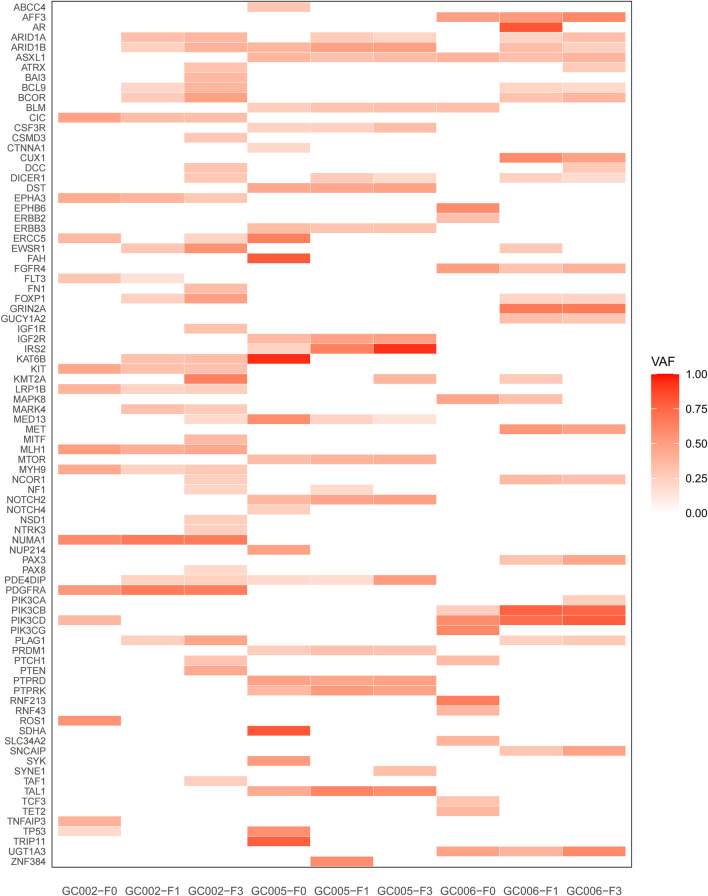
Heatmap of somatic mutation profiles across primary gastric tumors (F0) and their corresponding first- (F1) and third-generation (F3) PDX models. Each row represents a gene, and each column represents a sample (F0: primary tumor; F1/F3: first and third-generation PDX models). The color intensity reflects the VAF of somatic mutations, ranging from 0.00 (white) to 1.00 (dark red). Mutations are detected in 53 cancer-related genes across three representative gastric cancer cases (GC002, GC005, GC006).

The somatic mutation burden increased progressively across passages, with 64, 81, and 121 mutations identified in F0, F1, and F3, respectively. A mutation profile heatmap revealed that a core set of driver alterations was stably maintained across generations, whereas additional mutations emerged during serial passaging (Figure 5). Specifically, 28 genes—including *ARID1B, ASXL1, ERBB3, IGF2R, IRS2, KIT, PIK3CB,* and *PTPRK*—retained mutations across all three generations, reflecting the genomic stability of core driver events in PDX models. Notably, the variant allele frequencies (VAFs) of mutations in *ARID1B, IRS2, PIK3CB, PTPRK,* and *TAL1* increased consistently from F0 to F3, suggestive of clonal expansion under *in vivo* selective pressure.

Conversely, multiple F0-specific mutations—including those in *ABCC4, CTNNA1, ERBB2, EPHB6, FAH, NOTCH4, NUP214, PIK3CG, RNF213, RNF43, ROS1, SDHA, SLC34A2, SYK, TET2,* and *TRIP11*—were undetectable in later passages. This loss may reflect either true clonal extinction driven by selective pressures in the murine microenvironment or reduced detection sensitivity due to changes in tumor purity or sequencing coverage. *De novo* somatic mutations not present in F0 emerged in F1 and/or F3, including alterations in *ARID1A, BCL9, BCOR, DICER1, PLAG1,* and *SNCAIP*.

As shown in Table 1, ARID1A was wild-type in all F0 samples but acquired three mutations in F1 and six in F3. Similarly, ARID1B harbored a single mutation in F0 but expanded to 16 and 20 mutations in F1 and F3, respectively. Mapping of mutation sites revealed that most variants in both genes clustered within their N-terminal regions, which contain intrinsically disordered domains essential for protein–protein interactions (Supplementary Figure S1).

**TABLE 1:** Mutations in ARID1A and ARID1B in the F0, F1-PDX and F3-PDX models.

Sample ID	Tumour model	ARID1A	ARID1B
GC002	F0	NA	NA
	F1-PDX	P153A	P228S G278S C295Y 307_307del A329delinsAG G360S G396delinsGAA
	F3-PDX	P153A A226P P227Q 259_260del	P228S 269_270del G278S C295Y 307_307del A329delinsAG G360S A363S G396delinsGAA M479I M479L
GC005	F0	NA	P618R
	F1-PDX	P153A	G278S C295Y G396delinsGAA P618R
	F3-PDX	P153A	P618R
GC006	F0	NA	NA
	F1-PDX	P153A	P228S G278S C295Y 307_307del G396delinsGAA
	F3-PDX	259_260del	P228S 269_270del G278S C295Y 307_307del G360S G396delinsGAA M479L

NA, indicates “Not detected”.

### Prediction of potential deleterious mutations in gastric cancer

We identified 28 somatic mutations conserved between primary tumors and matched PDXs across three cases: GC002 (n = 9), GC005 (n = 14), and GC006 (n = 6). Among these, the *ASXL1* p. G652S variant was shared by GC005 and GC006, whereas the remaining mutations were case-specific. The overall conservation rate of somatic mutations between F0 and PDX models was 43.8% (28/64).

The functional impact of these 28 conserved mutations was assessed using four *in silico* prediction algorithms: SIFT, PolyPhen-2 HDIV, PolyPhen-2 HVAR, and MutationTaster. SIFT predicted 13 mutations as deleterious, PolyPhen-2 (both HDIV and HVAR) classified 12 as probably damaging, and MutationTaster predicted 16 as disease-causing (Table 2). Ten mutations were concordantly predicted as deleterious by all four algorithms. The genomic positions of these ten mutations are depicted in a lollipop plot (Supplementary Figure S2).

**TABLE 2:** The prediction of potential deleterious mutations among the 28 conserved mutations.

Genes	AA change	dbSNP ID	COSMIC ID	SIFT	Polyphen2_HVAR	Polyphen2_HDIV	MutationTaster
EPHA3	NM_005233: exon16, p.R914H, missense mutation	rs17801,309	COSM3736325	0.584, T	0.005, B	0.009, B	0.996, P
KIT	NM_000222: exon10, p.M541L, missense mutation	rs3822214	COSM28026	0.804, T	0.015, B	0.012, B	0.947, N
LRP1B^#^	NM_018557: exon35, p.A1912T, missense mutation	rs199906149	COSM5776518	0.003, D	0.996, D	1.000, D	1.000, D
MLH1	NM_001167619: exon17, p.Q460K, missense mutation	rs63750114	COSM6023788	0.948, T	0.114, B	0.536, P	0.999, D
MYH9	NM_002473: exon16, p.H615A, missense mutation	NA	NA	0.694, T	0.030, B	0.020, B	1.000, D
MYH9	NM_002473: exon2, p.R7R, synonymous mutation	NA	COSM1033855	0.077, T	0.019, B	0.226, B	1.000, D
NUMA1^#^	NM_001286561: exon19, p.R1667C, missense mutation	rs74985106	COSM5827038	0.003, D	0.931, D	1.000, D	0.999, D
NUMA1	NM_006185: exon15, p.A794G, missense mutation	rs3750913	COSM6030576	0.073, T	0.063, B	0.117, B	1.000, D
PDGFRA^#^	NM_006206: exon9, p.H425Y, missense mutation	rs759374919	COSM3917925	0.033, D	0.970, D	0.998, D	1.000, D
ARID1B	NM_017519: exon3, p.P618R, missense mutation	NA	NA	0.019, D	0.118, B	0.118, B	0.999, N
BLM	NM_000057: exon3, p.K116N, missense mutation	NA	NA	0.066, T	0.011, B	0.023, B	1.000, N
CSF3R	NM_000760: exon9, p.V352L, missense mutation	NA	NA	0.123, T	0.131, B	0.702, P	1.000, N
DST	NM_015548: exon23, p.V1127L, missense mutation	rs76257231	COSM4004102	0.237, T	0.967, D	0.984, D	1.000, N
ERBB3^#^	NM_001982: exon28, p.R1213W, missense mutation	NA	NA	0.000, D	0.975, D	0.999, D	0.952, D
IGF2R^#^	NM_000876: exon41, p.G2052R, missense mutation	NA	NA	0.002, D	1.000, D	1.000, D	1.000, D
MTOR^#^	NM_004958: exon34, p.K1606Q, missense mutation	NA	NA	0.002, D	0.987, D	0.991, D	1.000, D
NOTCH2^#^	NM_001200001: exon14, p.C772Y, missense mutation	NA	NA	0.000, D	1.000, D	1.000, D	1.000, D
PDE4DIP	NM_001198834: exon44, p.P2355H, missense mutation	rs113954821	COSM4142454	NA	NA	NA	NA
PRDM1	NM_182907: exon5 p.R591Q, missense mutation	rs940196192	COSM3619206	0.037, D	0.366, B	0.903, P	1.000, D
PTPRD	NM_002839: exon28, p.R995C, missense mutation	rs35929428	COSM3764024	0.096, T	0.071, B	0.321, B	0.000, P
PTPRK^#^	NM_002844: exon20, p.L988S, missense mutation	NA	NA	0.000, D	0.998, D	1.000, D	1.000, D
TAL1	NM_003189: exon2, p.E8A, missense mutation	NA	NA	0.000, D	0.028, B	0.077, B	0.991, N
PIK3CD	NM_005026: exon11, p.T456A, missense mutation	rs28730674	COSM3997716	0.257, T	0.001, B	0.000, B	1.000, N
ASXL1	NM_015338: exon12, p.G652S, missense mutation	rs3746609	COSM1716555	0.653, T	0.035, B	0.444, B	1.000, N
AFF3	NM_001025108: exon16, p.H914Q	rs117712488	COSM6352057	0.140, T	0.996, D	1.000, D	0.975, D
FGFR4	NM_022963: exon4, p.T179A, missense mutation	rs55675160	COSM5021091	0.279, T	0.186, B	0.525, P	1.000, N
PIK3CB^#^	NM_006219: exon20, p.F934L, missense mutation	NA	NA	0.001, D	0.991, D	1.000, D	1.000, D
UGT1A3^#^	NM_019093: exon1, p.R45W, missense mutation	rs45625338	COSM4989345	0.000, D	0.946, D	0.998, D	1.000, D

NA, indicates “not available”; # symbol indicates that the mutation was predicted as “D” by all four tools (SIFT, Polyphen2_HVAR, Polyphen2_HDIV, and Mutation Taster). SIFT: A lower score may indicate a greater likelihood of the SNP, causing structural or functional changes in the protein. “D” indicates “Deleterious”; “T” indicates “Tolerated”; Polyphen2_HVAR, and Polyphen2_HDIV: A higher score may indicate a greater likelihood of the SNP, causing structural or functional changes in the protein. “D” indicates “Damaging”; “B” indicates “Benign”; “P” indicates “Possibly damaging”; Mutation Taster: The prediction results indicate the impact on the protein sequence. The higher the score is, the more reliable the results (0–1); “P” indicates “Polymorphism automatic”, “N” indicates “Polymorphism”, and “D” indicates “Disease causing”.

Notably, four of these mutations—*LRP1B* p. A1912T, *PDGFRA* p. H425Y, *NUMA1* p. R1667C, and *UGT1A3* p. R45W—are catalogued in both dbSNP and COSMIC. Additionally, six novel deleterious mutations were identified in *MTOR* (p.K1606Q), *NOTCH2* (p.C772Y), *PTPRK* (p.L988S), *IGF2R* (p.G2052R), *ERBB3* (p.R1213W), and *PIK3CB* (p.F934L). To evaluate recurrence in gastric cancer, we queried the TCGA-STAD (Firehose Legacy) dataset (n = 441) via cBioPortal (https://www.cbioportal.org). Of these 10 genes, 8 (80%) were altered in ≥5% of TCGA-STAD cases, supporting their status as recurrently mutated genes in gastric adenocarcinoma (Supplementary Figure S3).

We further evaluated the impact of these ten mutations on protein stability using I-Mutant 2.0 and MUpro (Table 3). Both algorithms predicted decreased protein stability for nine of the ten mutations. Notably, *PTPRK* p. L988S, *PIK3CB* p. F934L, *LRP1B* p. A1912T, and *IGF2R* p. G2052R exhibited substantial destabilization, with ΔΔG values of −3.44 (I-Mutant)/−1.95 (MUpro), −2.70/−1.21, −1.41/−1.38, and −1.34/−0.76, respectively.

**TABLE 3:** Prediction of the protein stability of 10 potential deleterious mutations.

Genes	mRNA	AA change	I-mutant			MUpro	
			Stability	RI (0–10)	ΔΔG (Kcal/mol)	Stability	ΔΔG (Kcal/mol)
LRP1B	NM_018557	p.A1912T	Decrease	8	−1.41	Decrease	−1.38
PDGFRA	NM_006206	p.H425Y	Decrease	1	−0.68	Decrease	−0.24
NUMA1	NM_001286561	p.R1667C	Increase	0	0.08	Decrease	−0.15
MTOR	NM_004958	p.K1606Q	Decrease	6	−0.68	Decrease	−0.62
NOTCH2	NM_001200001	p.C772Y	Decrease	4	−0.49	Decrease	−0.64
PTPRK	NM_002844	p.L988S	Decrease	9	−3.44	Decrease	−1.95
IGF2R	NM_000876	p.G2052R	Decrease	8	−1.34	Decrease	−0.76
ERBB3	NM_001982	p.R1213W	Decrease	8	−0.44	Decrease	−0.60
UGT1A3	NM_019093	p.R45W	Decrease	7	−0.85	Decrease	−1.00
PIK3CB	NM_006219	p.F934L	Decrease	6	−2.70	Decrease	−1.21

RI, reliability index; ΔΔG, free energy value (mutated protein)-free energy value (wild protein) in kcal/mol; ΔΔG <0, decrease in stability; ΔΔG >0, increase in stability.

## Discussion

Patient-derived xenograft (PDX) models have emerged as powerful platforms for recapitulating the biological and genomic fidelity of human tumors, offering significant advantages over conventional cell line–based systems in preclinical oncology research. In this study, we successfully established seven F3-PDX models from 20 surgically resected gastric cancer specimens, yielding an engraftment success rate of 35%—substantially higher than previously reported rates of 15.1% and 24.4% (Kuwata et al., 2019; Choi et al., 2016). This improved efficiency likely stems from optimized tumor acquisition protocols, selection of treatment-naïve specimens, and preferential engraftment of tumors from anatomically favorable primary sites, all of which are known to influence PDX take rates (Katsiampoura et al., 2017; Byrne et al., 2017). Furthermore, we observed a progressive shortening of tumor latency across successive passages (F0 → F1 → F3), consistent with prior observations in gastric cancer PDX models (Zhu et al., 2015; Yao et al., 2022), suggesting adaptation and clonal selection during serial propagation.

Despite their growing utility, most existing gastric cancer PDX resources lack comprehensive molecular annotation, limiting their application in precision oncology and biomarker discovery. To address this gap, we performed whole-exome sequencing (WES) on three primary tumors (F0) and their matched PDX passages (F1–F3), enabling longitudinal tracking of somatic alterations throughout engraftment and *in vivo* expansion. This approach allowed us to distinguish tumor-intrinsic, stably conserved mutations from acquired or context-dependent variants, thereby refining the identification of putative driver events.

Our WES analysis of the three F0 tumors revealed somatic alterations in *IRS2*, *BLM*, *PDE4DIP*, *NUMA1*, *MYH9*, *TP53*, *PIK3CD*, *ERCC5*, and *ASXL1*. Notably, this mutational profile only partially overlaps with large-scale genomic studies of gastric adenocarcinoma. For instance, a large-scale genomic study of 529 Chinese gastric adenocarcinomas highlighted *TP53*, *ARID1A*, *LRP1B*, *PIK3CA*, and *CDH1* as top mutated genes (Yu et al., 2021). whereas another NGS-based analysis of 45 Chinese patients emphasized mutations in *MLL4*, *ERBB3*, *FBXW7*, *MTOR*, and *NOTCH1* (Pan et al., 2018). The discrepancies observed here are almost certainly attributable to our extremely limited sample size (n = 3). With such a small cohort, even moderately recurrent drivers (e.g., *ARID1A* or *CDH1*, altered in >20% of cases in larger datasets) may be missed by chance, while rare or passenger variants unique to individual tumors may appear spuriously enriched. Moreover, given the well-established molecular heterogeneity of gastric cancer—including chromosomal instability (CIN), microsatellite instability (MSI), Epstein–Barr virus (EBV)-positive, and genomically stable (GS) subtypes—our three cases may represent a non-representative subset, further skewing the observed mutational landscape. Consequently, the specific gene alterations identified in our primary tumors should be interpreted as hypothesis-generating rather than reflective of population-level recurrence patterns.

Nonetheless, the longitudinal design of our study provides a unique advantage: by tracking variant allele frequencies (VAFs) across F0 → F1 → F3 passages, we operationally classified somatic mutations into conserved (present in all passages) and acquired (emerging or expanding during passaging) categories (Figure 5). Among the 28 conserved mutations, several reside in canonical cancer driver genes—including *KIT, LRP1B, PDGFRA, ERBB3, MTOR, NOTCH2, PTPRD, PIK3CB,* and *PIK3CD*—suggesting their functional importance in maintaining core oncogenic programs. In contrast, early subclonal mutations such as *TP53, ERBB2, EPHA3,* and *SDHA* were progressively lost during PDX propagation, implying that the corresponding subclones either failed to engraft or became dispensable under murine selective pressures (Ben-David et al., 2017).

Strikingly, we observed the emergence of late-acquired variants—including *ARID1A/B, BCL9, BCOR, DICER1, KMT2A,* and *PLAG1*—which were undetectable in F0 but reached VAFs ≥15% by F3. This rapid clonal expansion strongly suggests positive selection within the murine microenvironment. Notably, *ARID1A* and *ARID1B* mutations showed progressive enrichment across passages. As core subunits of the SWI/SNF (cBAF) chromatin remodeling complex, loss-of-function alterations in these genes disrupt chromatin architecture, impair DNA repair, and promote epigenetic plasticity (Krishnamurthy et al., 2022; Liang et al., 2024). Such changes may facilitate metabolic reprogramming and immune evasion, conferring a fitness advantage during xenograft adaptation (Dong et al., 2022; Chen et al., 2023; Caumanns et al., 2025). Importantly, concurrent *ARID1A*/*ARID1B* mutations have been reported in gastric, endometrial, and liver cancers (Kelso et al., 2017) and may act synergistically to drive tumorigenesis, positioning *ARID1B* as a potential therapeutic vulnerability in *ARID1A*-deficient contexts (Helming et al., 2014).

Intriguingly, the *ARID1A* and *ARID1B* mutations in our models clustered within their N-terminal intrinsically disordered regions (IDRs)—a domain critical for cBAF condensate formation, chromatin targeting, and protein–protein interactions (Patil et al., 2023). Although these variants were classified as “benign” by conventional *in silico* predictors (e.g., SIFT, PolyPhen-2)—which are primarily trained on structured domains—recent evidence indicates that IDR mutations can subtly alter biophysical properties (e.g., charge, hydrophobicity, linear motifs) to disrupt higher-order biomolecular organization without affecting global protein stability (Garcia et al., 2022). The recurrent localization of these variants within the IDR, coupled with their progressive VAF increase across passages, strongly argues against them being neutral passengers. Instead, they likely fine-tune cBAF complex dynamics to enhance epigenetic adaptability—a mechanism distinct from complete loss-of-function and potentially less susceptible to negative selection.

To prioritize functionally relevant conserved mutations, we integrated predictions from SIFT, PolyPhen-2, and MutationTaster, identifying ten high-confidence deleterious variants. Acknowledging the limitations of *in silico* tools—including training bias toward common variants and inter-algorithm discordance (Donner et al., 2021; Wang et al., 2024) —we adopted a conservative consensus approach to minimize false positives. Subsequent protein stability analysis using I-Mutant 2.0 and MUpro (Capriotti et al., 2005; Cheng et al., 2006) highlighted four mutations with pronounced destabilizing effects: *PTPRK* (p.L988S), *PIK3CB* (p.F934L), *LRP1B* (p.A1912T), and *IGF2R* (p.G2052R).

*PTPRK*, a candidate tumor suppressor encoding a receptor-type tyrosine phosphatase, harbors its L988S mutation within the catalytically active D1 domain. This alteration may impair dephosphorylation of oncogenic substrates, potentially acting as a gain-of-function driver. Similarly, the *PIK3CB* p. F934L mutation resides in the same kinase domain as the experimentally validated oncogenic E1051K variant (Whale et al., 2017), which enhances p110β catalytic activity and drives PI3K signaling independent of upstream activation. Given that p. F934L similarly localizes to this critical functional region, it represents a compelling candidate for isoform-selective PI3Kβ inhibition. *LRP1B*, one of the most frequently mutated tumor suppressors in gastric cancer (Wang et al., 2023), carries the A1912T substitution—a variant documented in COSMIC and dbSNP but lacking functional validation. Finally, the *IGF2R* G2052R mutation lies within the 14th extracellular repeat domain responsible for IGF-II binding (Brown et al., 2008); its presence may compromise ligand sequestration, thereby attenuating *IGF2R*’s tumor-suppressive function (Martin-Kleiner and Koraljka, 2010).

Collectively, these destabilizing mutations likely perturb key cellular processes—including signal transduction, proliferation, and apoptosis—and may serve as actionable targets. Of particular therapeutic interest is *PIK3CB* p. F934L, which localizes to the catalytic core and may confer resistance to pan-PI3K inhibitors while remaining sensitive to β-isoform–selective agents (e.g., GSK2636771 or AZD8186). To validate this hypothesis, we are currently generating isogenic cell lines and transgenic mouse models expressing this variant to assess drug sensitivity. Concurrently, we plan to use CRISPR interference (CRISPRi) to knock down mutant *ARID1B* in PDX-derived organoids, evaluating its impact on SWI/SNF complex integrity, chromatin accessibility, and transcriptional networks driving gastric cancer progression.

Finally, while our KEGG pathway enrichment analysis of the 53 somatic mutations in F0 tumors indicated significant enrichment in “Pathways in cancer” and the “PI3K-Akt signaling pathway” (Supplementary Table S4), these findings must be interpreted with caution. The extremely small input gene set (n = 53) and limited cohort size (n = 3) severely constrain statistical power and increase susceptibility to overrepresentation artifacts—particularly for broad, highly connected pathways like PI3K-Akt. Thus, these results should be regarded as exploratory and require validation in larger, molecularly stratified cohorts.

In summary, our study not only demonstrates the feasibility of establishing high-fidelity gastric cancer PDX models with enhanced engraftment efficiency but also provides a longitudinally annotated molecular resource that distinguishes stable driver events from microenvironment-driven adaptations. By integrating genomic tracking, *in silico* prioritization, and functional hypotheses, we lay the groundwork for future mechanistic studies and biomarker-driven therapeutic development in gastric cancer.

## Conclusion

In conclusion, our study establishes a molecularly annotated panel of gastric cancer PDX models with an enhanced engraftment rate and demonstrates the power of longitudinal genomic tracking to distinguish tumor-intrinsic driver alterations from microenvironment-driven adaptive changes. By integrating exome sequencing across serial passages, functional prediction, and pathway analysis, we identify conserved deleterious mutations—including *PIK3CB* p. F934L and *PTPRK* p. L988S—as potential therapeutic targets, and reveal progressive selection of *ARID1A/B* mutations in intrinsically disordered regions that may fine-tune SWI/SNF complex function during xenograft adaptation. Although limited by a small cohort size, our findings provide a robust preclinical framework for validating genotype–phenotype relationships and advancing precision oncology strategies in gastric cancer.

## Data Availability

The original contributions presented in the study are publicly available. Raw sequence data reported in this study have been deposited in the Genome Sequence Archive (GSA) at the National Genomics Data Center, China National Center for Bioinformation / Beijing Institute of Genomics, Chinese Academy of Sciences, under accession numbers HRA016162 (GSA‐Human) and CRA037299 (GSA). The datasets are publicly accessible at https://ngdc.cncb.ac.cn/gsa-human and https://ngdc.cncb.ac.cn/gsa, respectively.
