# Supplementary material for: Identification of potentially deleterious mutations in gastric cancer using patient-derived xenograft models
Source: Front Genet. 2026 Jan 29;16:1571535. doi: 10.3389/fgene.2025.1571535 (PMC12895051; doi:10.3389/fgene.2025.1571535)
Supplement: Supplementary file 1 [file Supplementaryfile1.zip › 20260103_SupMaterial/Supplementary Figure S1.pdf]

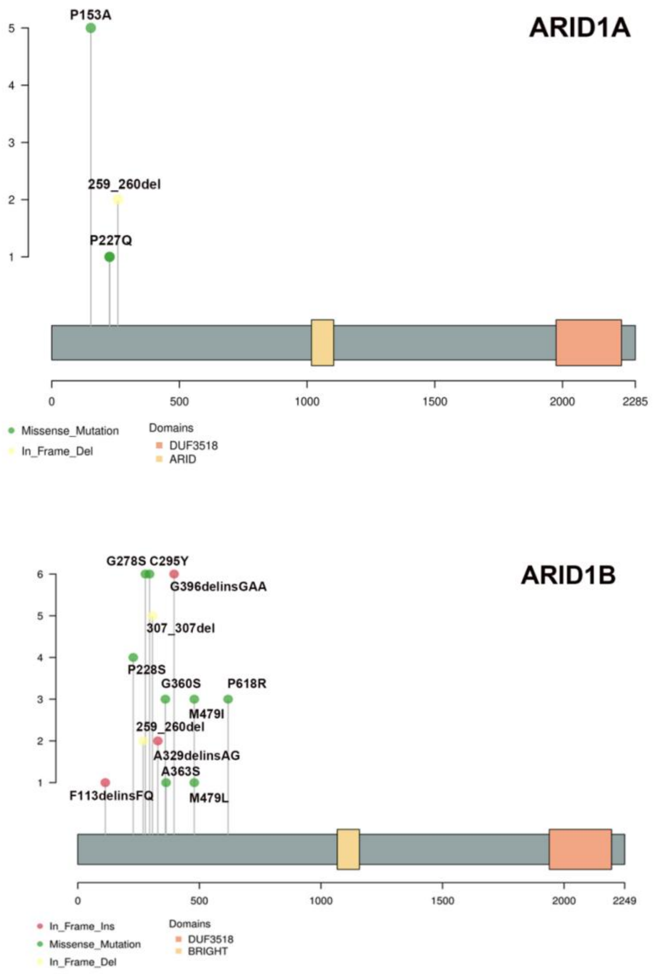

Supplementary Figure S1. Recurrent somatic mutations in ARID1A and ARID1B cluster within N-terminal intrinsically disordered regions across PDX passages.
