# Supplementary material for: Identification of potentially deleterious mutations in gastric cancer using patient-derived xenograft models
Source: Front Genet. 2026 Jan 29;16:1571535. doi: 10.3389/fgene.2025.1571535 (PMC12895051; doi:10.3389/fgene.2025.1571535)
Supplement: Supplementary file 1 [file Supplementaryfile1.zip › 20260103_SupMaterial/Supplementary Figure S3.pdf]

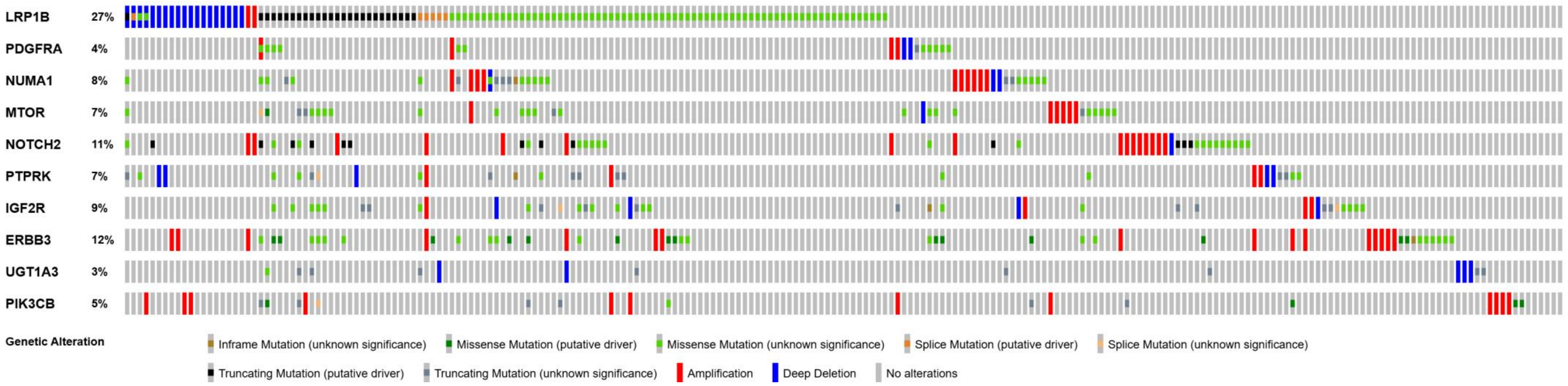

Supplementary Figure S3. The frequencies of 10 potential deleterious mutations in the TCGA-STAD Firehose Legacy dataset (n = 441) were analyzed using cBioPortal.
